# Standing genetic variation as a major contributor to adaptation in the Virginia chicken lines selection experiment

**DOI:** 10.1186/s13059-015-0785-z

**Published:** 2015-10-01

**Authors:** Zheya Sheng, Mats E. Pettersson, Christa F. Honaker, Paul B. Siegel, Örjan Carlborg

**Affiliations:** Division of Computational Genetics, Department of Clinical Sciences, Swedish University of Agricultural Sciences, Uppsala, Sweden; Department of Animal and Poultry Sciences, Virginia Polytechnic Institute and State University, Blacksburg, VA USA; Present address: Department of Medical Biochemistry and Microbiology, Uppsala University, Uppsala, Sweden

**Keywords:** Selective-sweep mapping, Genome-wide association analysis, Advanced Intercross Lines, Allele-substitution effects, Selection-response, Experimental evolution, Body-weight, Chicken, Virginia lines

## Abstract

**Background:**

Artificial selection provides a powerful approach to study the genetics of adaptation. Using selective-sweep mapping, it is possible to identify genomic regions where allele-frequencies have diverged during selection. To avoid false positive signatures of selection, it is necessary to show that a sweep affects a selected trait before it can be considered adaptive. Here, we confirm candidate, genome-wide distributed selective sweeps originating from the standing genetic variation in a long-term selection experiment on high and low body weight of chickens.

**Results:**

Using an intercross between the two divergent chicken lines, 16 adaptive selective sweeps were confirmed based on their association with the body weight at 56 days of age. Although individual additive effects were small, the fixation for alternative alleles across the loci contributed at least 40 % of the phenotypic difference for the selected trait between these lines. The sweeps contributed about half of the additive genetic variance present within and between the lines after 40 generations of selection, corresponding to a considerable portion of the additive genetic variance of the base population.

**Conclusions:**

Long-term, single-trait, bi-directional selection in the Virginia chicken lines has resulted in a gradual response to selection for extreme phenotypes without a drastic reduction in the genetic variation. We find that fixation of several standing genetic variants across a highly polygenic genetic architecture made a considerable contribution to long-term selection response. This provides new fundamental insights into the dynamics of standing genetic variation during long-term selection and adaptation.

**Electronic supplementary material:**

The online version of this article (doi:10.1186/s13059-015-0785-z) contains supplementary material, which is available to authorized users.

## Background

Adaptation is a dynamic evolutionary process where populations improve their fitness by accumulating beneficial alleles at loci controlling adaptive phenotypes. The polymorphisms contributing to adaptation can either be present as standing genetic variation at the onset of selection or emerge through mutations. A longstanding challenge in quantitative and evolutionary genetics has been quantification of the relative contributions from standing and emerging variation to long-term selection response [1, 2]. While such results are very difficult to obtain in studies of natural populations, artificial selection provides an approach to study the origin and fate of beneficial mutations during adaptation [2].

Subjecting populations to artificial selection provides an accelerated evolutionary process that may result in extreme phenotypes with accompanying changes across the genome [2–4]. Using such experiments, the contribution by mutational variance to the evolution of quantitative traits can be quantified by, for example, measuring the release of genetic variance during selection experiments from an inbred founder population [5]. Estimating the contribution from standing genetic variation to long-term selection response is, however, more complex. Whereas short-term contributions can be estimated based on the immediate selection response in a selection-experiment starting from outbred founders, long-term contributions of standing variation are more difficult to estimate due to confounding with effects of mutations that emerge over time. Other approaches are therefore needed to disentangle the contributions of standing variation from other sources of selectable additive variation such as new mutations.

Selection on variants that have been present in a population for some time prior to the onset of selection can result in a different genomic footprint of selection than that of selection on new mutations. The term ‘soft’ sweep has been introduced to distinguish the patterns resulting from selection on standing genetic variation or recurrent new mutation from the ‘hard’ sweep patterns observed after selection on single new mutations [6–9]. The presence of soft sweeps in the genome of a selected population therefore suggests that standing genetic variation has been important for adaptation. Traditional selective-sweep studies are, however, unable to distinguish between fixations that are due to selection and those due to other population-genetics force, such as drift. Here, we report results for associations of previously identified soft selective sweeps. Namely, candidate regions that contain adaptive standing genetic variants, in a long-term selection experiment for 56-day high and low body weights in chickens. Individual loci containing selected standing genetic variants are identified and their individual and joint contributions to adaptation quantified.

In this study, we utilise an experimental population developed by bi-directional selection from a common, segregating founder-population to estimate the contribution of standing genetic variation to long-term selection-response. For several reasons, this population is highly useful for separating the contributions from standing genetic variation and new mutations even when molecular data on the base population is missing [3, 4]. First, as the population has been subjected to long-term bi-directional selection from a common base population, it is highly useful for identifying selective sweeps. In particular, this is because fixation for alternative alleles across genomic regions in the divergently selected lines is expected to be common when selecting on standing variants, but rare when selecting on novel mutations. Second, as selection was on a single trait it is possible to evaluate the contribution of individual sweep regions to selection response by testing for association between the alleles that were fixed in the divergent populations and the selected trait in an intercross population derived from them. Here, we evaluated the genetic contributions of a large collection of such divergently fixed selective sweeps [3] (that is, standing genetic variants) to the selected trait in an intercross population derived from the selected lines to independently predict their contribution to selection response. This provides insights into the dynamic processes involved in shaping the genetics of complex traits during adaptive evolution.

**Fig. 1:**
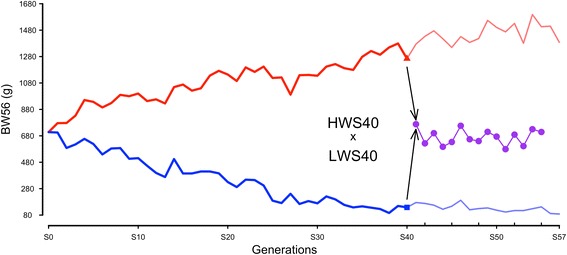
Body weights at 56 days of age in the Virginia weight selected and Advanced Intercross Lines. Average body weights per generation are provided for females in the high and low body-weight selected lines and as sex-averaged weights in the Advanced Intercross Line. BW56: 56-day body weight

The base population for the Virginia lines was established in 1957 by intercrossing seven partially inbred lines of White Plymouth Rock chickens. The genetic variation entering the population thus represents a sample of the polymorphisms present when these partially inbred lines were founded. Since the founder population was established, the high (HWS) and low (LWS) body-weight lines have been bred with one new generation per year by single-trait, bi-directional selection for 56-day body weight [10–12]. The response to selection has progressed steadily throughout the experiment, resulting in an eight-fold difference in 56-day body weight after 40 generations of selection and currently, in the 57th generation, there is a 16-fold difference between the lines (Fig. 1). Genome-wide comparisons between the divergently selected lines have identified more than 100 candidate adaptive sweeps between them [3, 4]. The contribution to selection response of these candidate selective-sweep regions that originate from the standing genetic variation is still unknown. Here, we identified which of these candidate selective-sweeps contributed to adaptation and estimated their individual and joint contributions to the adaptive trait.

This study describes a genome-wide approach to explore the contributions from a large number of selective-sweeps [3] to selection-response and adaptation in the Virginia chicken lines. A deep intercross population from HWS and LWS from generation S40, and genotyping and phenotyping a large F_15_ Advanced Intercross Line (AIL) for the selective-sweep regions, allowed us to estimate the contribution of these sweeps to the adaptive trait: 56-day body weight. We found that the standing genetic variation at a large number of loci in the base population made a major contribution to the selectable additive variance during adaptation. Further, the results also show that fixation across several loci has been an important dynamic property in the selection on the highly polygenic genetic architecture of the selected trait in the base-population. Together they suggest that fixation across many loci containing variants present in the base population has been an important contributor to the gradual, continued, long-term response to selection in the Virginia lines.

## Results

**Fig. 2:**
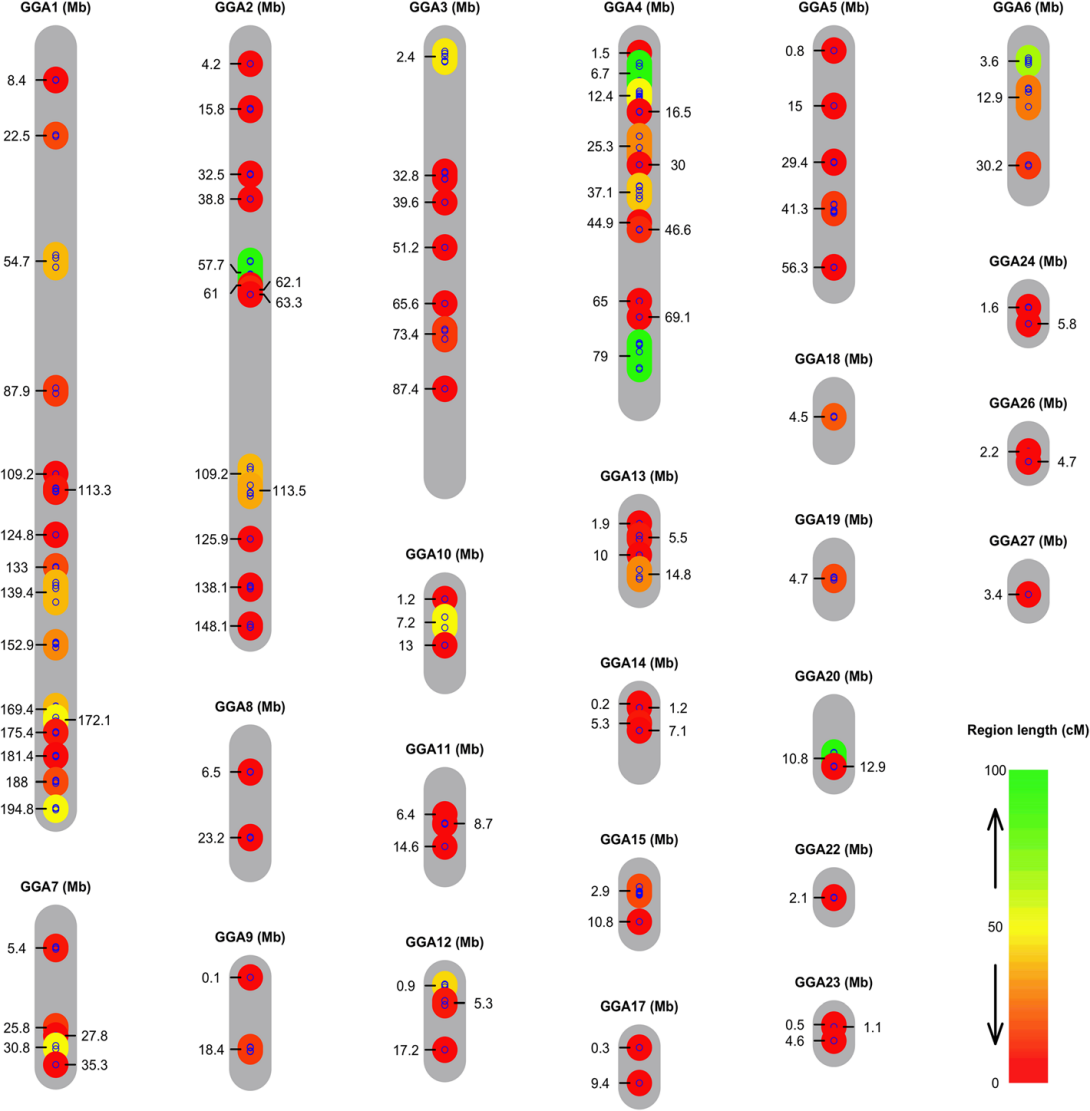
Genomic distribution of the selective sweeps. The grey bars represent chromosomes with their lengths in Mb on the November 2011 (galGal4) genome assembly. The small blue dots indicate the locations of the 252 genotyped markers that passed quality control. The coloured bars connecting the dots on the chromosomes illustrate the 99 independently segregating regions that were tested for association with 56-day body weight in the F_15_ generation of the Virginia Advanced Intercross Line. The centre of each selective sweep is indicated by its physical position in Mb. The colour of the bars indicate their genetic map lengths (cM, Haldane) in the F_15_ generation and the extension of their physical map lengths (Mb)

Evaluated were the contribution of 106 selective sweeps, where the high and low weight selected Virginia lines were fixed for alternative alleles, to selection response [3, 4]. Using genotypes for 252 markers in these sweeps, 99 clusters of markers that segregated independently were identified in the F_15_ generation of the AIL. Thirty-eight of these regions were covered by a single marker and 61 regions by multiple markers. The physical length of the regions covered by multiple markers was in the range of 0.02 to 6.7 Mb and were distributed across most autosomes in the chicken genome (Fig. 2; Additional file 1: Table S1).

### Many adaptive selective sweeps have contributed to selection-response

To estimate how many of the independently segregating regions contributed to 56-day body weight in the AIL F_15_ generation, we initially selected one representative marker from each of the 99 targeted regions via a within-region, backward-elimination analysis. Then, an across-region analysis was performed to identify the set of regions that jointly contributed to the adaptive trait. A multi-locus, backward-elimination analysis was used where the final set of loci were selected at 5 % and 20 % false discovery rate (FDR) thresholds [13, 14]. The potential influences of population structure were controlled using bootstrapping [15]. Table 1 summarises the loci associated with 56-day body weight at 5 % and 20 % FDR significance thresholds. Many of the 99 evaluated sweeps were associated with 56-day body weight in the F_15_ AIL, 8 at a 5 % and 16 at a 20 % FDR. Thus, we confirmed that a large number of the candidate selective sweeps identified previously [3] were adaptive selective sweeps. Further, we could also show that an important dynamic feature during selection on this highly polygenic genetic architecture was fixation of several standing variants within the divergent lines after 40 generations of selection.

**Table 1:** Genetic effects of selective sweeps associated with 56-day body weight in the Virginia Advanced Intercross Line. Estimates are provided for one marker in each associated sweep. Genetic effects were estimated in generation F_15_ of the AIL

GGA	Position^a^ (Mb)	Marker	Additive^b^ (a ± SE)	Sign^c^	RMIP^d^ (FDR 5/20 %)	Sign^e^
1	87	rs13899455	−22.7 ± 6.2	2.4 × 10^−4^	0.63/0.71	5 %
1	133	rs13942473	16.5 ± 8.0	3.9 × 10^−2^	0.38/0.59	20 %
1	142	rs15448487	16.5 ± 6.8	1.5 × 10^−2^	0.37/0.65	20 %
1	169	rs14916997	28.9 ± 6.3	6.0 × 10^−6^	0.93/0.98	5 %
2	61	GGaluGA149337	25.8 ± 7.9	1.2 × 10^−3^	0.75/0.85	5 %
2	112	rs15143460	23.8 ± 6.9	6.4 × 10^−4^	0.55/0.66	5 %
2	148	rs15158686	19.0 ± 6.3	2.8 × 10^−3^	0.76/0.86	5 %
3	34	rs15321683	24.6 ± 6.0	4.7 × 10^−5^	0.65/0.73	5 %
3	75	GGaluGA228961	15.5 ± 6.8	2.2 × 10^−2^	0.28/0.47	20 %
4	2	rs14417942	14.5 ± 6.7	3.0 × 10^−2^	0.31/0.48	20 %
4	12	GGaluGA246087	17.8 ± 6.6	7.3 × 10^−3^	0.37/0.51	20 %
4	45	rs15560796	14.5 ± 6.6	2.8 × 10^−2^	0.39/0.55	20 %
4	82	rs14498744	−31.3 ± 6.6	2.0 × 10^−6^	0.94/0.96	5 %
10	9	GGaluGA068581	11.3 ± 6.4	8.0 × 10^−2^	0.35/0.52	20 %
13	10	rs14059068	16.6 ± 6.3	8.6 × 10^−3^	0.42/0.61	20 %
23	5	rs15205573	18.5 ± 6.6	5.4 × 10^−3^	0.49/0.75	5 %

GGA: Gallus gallus autosome

^a^November 2011 (galGal4) assembly

^b^Additive genetic effect ± Standard Error estimated in model (C) or (D)

^c^Significance for additive genetic effect in model including all loci significant at 20 % FDR

^d^Resample Model Inclusion Probability [15] using 5 % or 20 % FDR threshold [13]

^e^Significance thresholds 5/20 % FDR that the marker was selected at with RMIP >0.46 in after Bagging procedure [15] to correct for population structure

### The individual adaptive selective sweeps have small allele-substitution effects

**Fig. 3:**
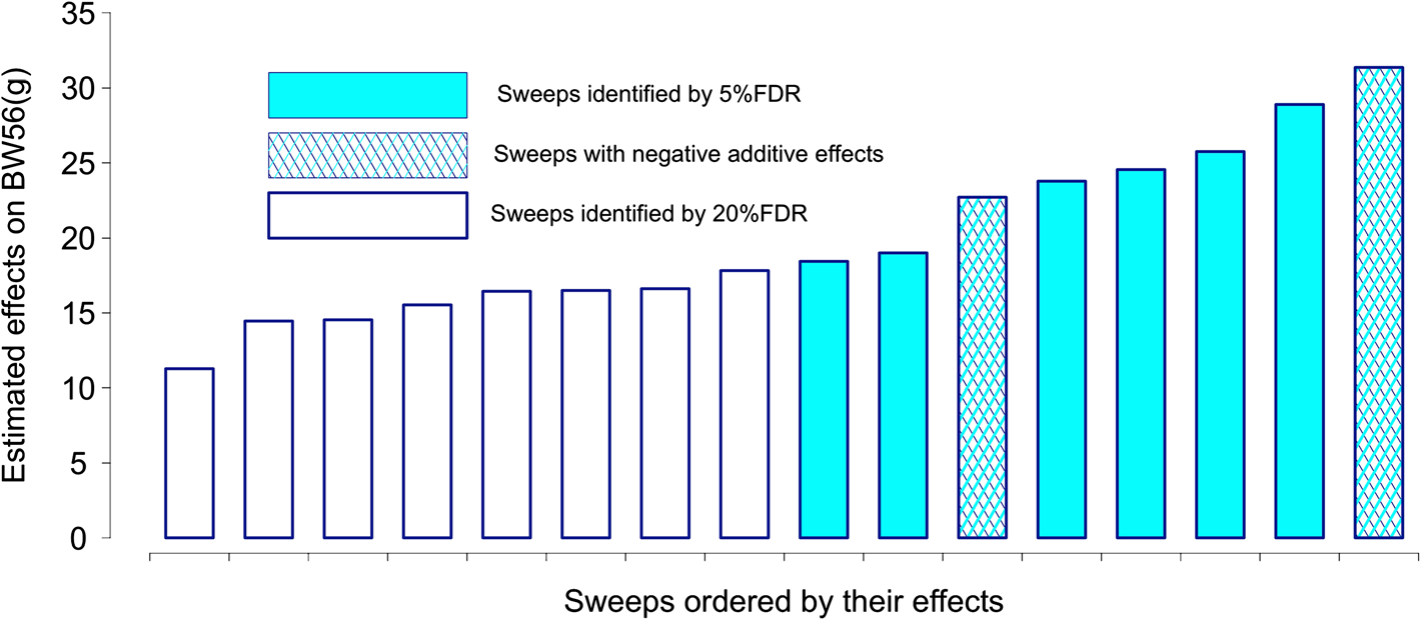
Allele-substitution effects of selective sweeps associated with 56-day body weight (BW56) in the Virginia Advanced Intercross Line. The effects were estimated in the F_15_ generation of the AIL. Coloured bars indicate for selective sweeps with associations at a 5 % FDR and white bars selective-sweeps with associations at a 20 % FDR. Solid coloured bars indicate selective sweeps where the HWS derived allele increases body weight. Hashed coloured bars indicate selective-sweeps where the LWS derived allele increase body weight, that is, regions that are transgressive

To estimate the contribution by the individual adaptive sweeps to the selected trait, additive, allele-substitution effects were estimated using a multi-locus association analysis. We found that the additive effects of the individual loci were generally small, and no individual locus had an additive allele-substitution effect greater than 29 g (or 0.2 σ_P_) for the selected trait (Table 1; Fig. 3). The effects were similar for most of the loci when estimated in the F_15_ population (Fig. 3a). Most of the HWS derived alleles increased weight, however, two regions also had transgressive effects on the trait – that is, that an allele inherited from the LWS increased weight. The standing genetic variation in the base population for the Virginia chicken lines thus has contributed with alleles of small effect across a highly polygenic genetic architecture.

### Larger selective sweeps have stronger effect on the selected trait

Larger selective sweeps are expected to contain polymorphisms with stronger effects because they are expected to be fixed more rapidly [3]. We tested this hypothesis by evaluating whether there was a difference in the lengths (in Mb) between sweeps with and without significant associations to the selected trait in the AIL F_15_ generation. We found a significant difference in the lengths between these two groups (2.1 Mb vs. 0.7 Mb; *P* = 0.02; one-sided *t*-test). This shows that the length of the identified selective sweeps is significantly associated with the effect of the genetic variant, or variants, located in that sweep.

### Large contribution by standing genetic variants to long-term selection response

In the AIL F_15_ population, none of the intercross chickens were HWS/HWS or LWS/LWS homozygous across all 16 regions. We therefore instead predict the joint contributions of the adaptive selective sweeps to the divergence between the lines based on the average allele-substitution effect for HWS alleles across the selective sweep regions shown to contribute to 56-day body weight. The average estimates for the sweeps that were significant at 5/20 % FDR were 15.7 ± 2.5 (*P* = 6.7 × 10^−10^) /16.9 ± 1.6 (*P* <1 × 10^−16^) g, respectively. The predicted total contribution of these regions to the 1,242 g line-difference between HWS40 and LWS40 (Table 2) are then 16 HWS alleles × 15.7 g = 251 g (20.2 %) for the 5 % set, and 32 HWS alleles × 16.9 g = 542 g (43.6 %) for the 20 % FDR set. Both of these estimates are biased downwards due to the inclusion of the two transgressive selective-sweep regions in the analysis. Thus, the total contribution by the adaptive selective sweeps from the standing variation in the base population to adaptation is likely to be at least 40 % of the total realised response at generation 40.

**Table 2:** Summary statistics for the body weights for the Advanced Intercross Line population. The AIL was bred between founders from generation 40 of the high (HWS) and low (LWS) selected lines

Generation	BW56 (mean/σ_P_)
F_0_ (HWS40)	1,412/125
F_0_ (LWS40)	170/47
F_1_-F_15_	672 [569–756]/148 [115–169]

BW56: 56-day body weight; σ_P_: Sex-averaged residual phenotypic standard deviation

### Contribution by standing genetic variation to the selectable additive genetic variance

The next step was to estimate the contribution of the associated sweeps to the genetic variance in the F_15_ generation of the AIL. The narrow-sense heritability for 56-day weight in the F_15_, estimated based on the pedigree kinship [16], was h^2^_BW56_ = 0.46. The eight regions associated at the more stringent 5 % FDR threshold together explained nearly one-fifth of the residual phenotypic variance in 56-day body weight (19.1 % of residual σ^2^_P_). When we included the eight additional regions selected at a 20 % FDR, the estimate increased to 23.4 % of σ^2^_P_. Thus, the associated selective-sweep regions contributed to approximately half of the additive genetic variance in this population (41.5 % and 50.9 % of h^2^_BW56_ for regions significant at 5/20 % FDR, respectively). Accordingly, the confirmed selective sweeps have been major contributors to the selectable additive genetic variance during this selection experiment. Further, this result also illustrates that fixation at these adaptive selective-sweep regions depleted about half of the total additive genetic variance for 56-day body weight during the first 40 generations of divergent selection.

### Estimating the contribution by adaptive selective sweeps to the additive genetic variance in the base population at the onset of selection

The joint contribution by the 16 associated selective sweeps to the additive genetic variance in the base population is dependent on their allele frequencies at onset of selection. These frequencies are, however, unknown and cannot be empirically determined as no biological samples were collected from the Viriginia lines prior to generation 40. Here, we therefore estimate their contribution to the additive genetic variance in the base population based on theoretically assumed allele frequencies.

We first assumed that each individual locus had the same allele frequency in the base population as in the AIL F_15_ generation, where the HWS alleles were present at frequencies were in the range of 0.2 to 0.8 (Additional file 1: Table S1). By comparing the total additive genetic variation in the base population obtained shortly after onset of selection [17] to the amount of additive genetic variance contributed by the adaptive selective sweeps in the AIL F_15_, we estimate that the additive genetic variance contributed by the sweeps corresponds to 86 % of that present in the base population (Table 3).

**Table 3:** Summary of estimates of genetic parameters in the Virginia lines. Estimates are provided for the base population of the Virginia chicken lines (P_0_) and the F_15_ generation from the Advanced Intercross Line population (F_15_)

Generation	BW56 (mean/σ_P_)	h^2^	h^2^_sweeps_ (5/20 % FDR)	σ_Atot_	σ_Asweeps_ (5/20 % FDR)
P_0_	797/120	0.30	–	36.0	–
F_15_	594/132	0.46	0.19/0.23	60.7	25.2/30.9

P_0_: Base population for Virginia lines; BW56: 56-day body weight; σ_P_: Sex-averaged residual phenotypic standard deviation; h^2^: narrow-sense heritability estimated based on realised selection-response (P_0_; [37]) or pedigree (F_15_); h^2^_sweeps_: heritability contributed by sweeps associated with BW56 at 5/20 % FDR thresholds; σ_Atot_: Total additive genetic standard deviation; σ_Asweeps_: Additive genetic standard deviation due to selective sweeps associated with BW56 at 5/20 % FDR thresholds

The base population was founded by intercrossing seven partially inbred chicken lines from the same breed. Using simulations, we then also evaluated the expected contribution by the sweeps at two theoretical frequency distributions representing scenarios where: (1) the seven intercrossed lines contributed randomly fixed adaptive alleles from the White Plymouth Rock breed (that is, a uniform distribution between 1/7-6/7); and (2) only one of the founder lines contributed the adaptive allele (that is, all loci had a frequency of 1/7). Under the assumption of randomly distributed adaptive alleles across the founder lines, the contribution was estimated to fall between 75 % and 92 %, which was very similar to the estimate based on the AIL F_15_ frequencies. The more conservative estimate, based on the assumption that only one founder line contributed the adaptive allele, was slightly lower at 50 %. Together, all results suggest that the adaptive selective sweeps identified and confirmed here represented a considerable portion of the standing genetic variation in the base population and was also an important contributor to the gradual long-term selection response in the Virginia chicken lines.

## Discussion

This study utilises our ability to differentiate the genomic patterns resulting from selection on standing genetic variation from those that emerge from selection on new variations [6–9]. In earlier work, we showed that soft selective sweeps are in a majority among the divergent fixations in the bi-directional Virginia lines selection experiment [3]. By testing for associations between these sweeps and the selected trait in a population where they segregate independently, we show both that a large number of standing genetic variants made individually significant contributions to the selected trait, and that their divergent fixation made a large contribution to the realised long-term selection response.

### Many selective sweeps associated with 56-day body weight

In total, 8/16 candidate selective-sweep regions were associated with 56-day body weight at 5/20 % FDR. In the original selective-sweep mapping study [3], simulations predicted that no less than 40 %, but most likely considerably more than half of the candidate selective sweeps originating from the base population would result from selection rather than drift. In light of results reported here, this theoretical prediction still appears realistic. First, we confirmed that the genetic architecture of 56-day weight in the base population was highly polygenic and that the contributions by individual loci were small. Also, quantitative genetics estimates were in agreement with this because they also predicted that the confirmed sweeps did not represent all of the standing additive genetic variance in the base population. As the power to detect loci with small individual effects was limited in the association analysis of only 800 F_15_ individuals, it is likely that several loci with similar or smaller effects than those confirmed here contributed to the trait but remained undetected. Second, although the current study covered 99 candidate selective sweeps, it was only a sample of all candidate selective sweeps present in the Virginia lines. Accordingly, we would expect to have missed some soft sweeps in the earlier mapping study [3] since it was based on medium-density 60 k SNP-chip genotyping rather than whole-genome re-sequencing. Also, the current association study was restricted as some candidate selective-sweep regions [3] were missing because of failed genotyping, as well as the effects of the tested regions here could not be separated due to the limited resolution in the AIL F_15_generation. Obtaining a complete understanding of the genetic architecture of selection response due to standing variation in the base population of the Virginia chicken lines will require additional candidate selective sweeps.

### Base-population alleles contribute to selection response via small individual genetic effects

The individual allele-substitution effects for the confirmed adaptive selective-sweep regions were small (Fig. 3). No individual locus had an allele-substitution effect greater than 29 g (0.2 residual σ_P_; Fig. 3; Table 1) corresponding to a contribution of less than 3 % of the residual σ^2^_P_ despite the average minor allele frequencies of the associated sweeps being rather high (average MAF = 0.38; Additional file 1: Table S1).

It follows the expectation from theoretical studies involving soft sweeps that most alleles originating from the standing genetic variation had small effects. The probability of fixation for alleles with small effects is higher when selection is intense and acts on standing genetic variation than on a new mutation as there is a higher likelihood that a weakly selected new mutation will be lost due to genetic drift [8].

### Fixation of several standing genetic variants was important for long-term selection response

Here, we empirically confirm that the genetic architecture of body weight in the base population of the Virginia lines was highly polygenic and that fixation of standing genetic variants at several loci across the genome has been an important mechanism underlying the rapid selection response leading to the phenotypic divergence between the divergently selected lines. Although our work is performed in the context of an artificial selection experiment, these results have interesting implications for the general discussion on how selection shapes phenotypic variation within and between populations. Our finding that rapid adaptation can proceed through fixation of standing genetic variants at several loci across a complex, multi-locus genetic architecture presents an alternative to the recently proposed polygenic adaptation model that suggests that adaptation proceeds through subtle frequency shifts across many loci [18]. Our results presents empirical evidence that clearly illustrates the value to, in addition to the ‘hard sweep’ and ‘polygenic’ models, also consider a ‘soft sweep’ adaptive model based on rapid fixation of standing genetic variants across a polygenic genetic architecture.

### The alleles originating from the standing genetic variation in the base population make a large joint contribution to the additive variance and selection response

The confirmed adaptive sweeps contributed about 40 % of the realised selection response and half of the total additive genetic variance in the F_15_ population. A significant portion of the phenotypic divergence between the lines was thus due to alleles originating from the standing genetic variation in the base population and for which the lines are now fixed for alternative alleles. This clearly illustrates the importance of the standing genetic variation in the base population for the gradual, long-term response to selection observed throughout this experiment.

Overall, our findings are consistent with those reported for another long-term selection experiment: the Illinois corn selection lines [19, 20], in particular the highly polygenic genetic architectures with many loci of small individual effects as well as the presence of transgressive loci. This suggests that the selection on highly polygenic genetic architectures, where many loci make minor contributions rather than on emerging mutations with large effects, has been important for the long-term success of these selection experiments. Such studies will continue to deliver valuable insights not only to the general features of polygenic architectures contributing to morphological traits in vertebrates, but also the basic processes involved in accelerated evolution and adaptation.

### On the presence of several transgressive sweeps

Two of the confirmed selective sweeps were transgressive, that is, the allele inherited from the low-weight selected line increased weight in the AIL F_15_ generation. Similar findings have also been reported in studies of other artificially selected populations (see, for example, [19, 21]), suggesting that the presence of transgressive alleles is to be expected in the analyses of highly selected lines.

Our observation is consistent with the theoretical work by Robertson, who in 1960 showed that alleles of small effect can be fixed by chance in the opposite direction to that of selection [22]. As the effects of the transgressive loci are not small in comparison to the other loci detected in this population, other alternative explanations should also be considered in future studies of these loci. One possibility is epistasis, where the effects of the alleles that are found to be transgressive will depend on the genetic background in the lines where they were fixed. As the genetic background in the AIL will be different from that of the selected lines, the alleles may have had smaller (or even opposite) effects at their time of fixation. As epistasis has been reported to be an important contributor to the selection response in these lines [23, 24], this potential explanation deserves particular attention in subsequent studies. Another potential explanation is fixation due to ‘hitch-hiking’ with beneficial alleles. This explanation is supported by the results from earlier analyses of an F2 intercross between the divergently selected Virginia lines. There it was observed that alleles affecting weight are distributed throughout a large part of the genome (see, for example, Fig. 3 in [25]), suggesting that it is not an unlikely event that linkage to multiple beneficial alleles might have contributed to the fixation of one, or several, of the transgressive alleles. Further, although the primary selection criterion has been 56-day body weight, pleiotropic selection for fitness has played a significant role while breeding the lines. The low-weight line has reached a plateau that is essentially physiological from getting too small, and further some of the birds do not reach sexual maturity due to being anorexic [10]. In the high-weight line, the birds are feed-restricted commencing at selection age, beginning at generation 18, to maintain reproductive fitness. Hence, it is also feasible that some of the transgressive alleles might have been fixed in the lines due to beneficial, pleiotropic effects on viability.

### Other sources of selectable additive variation in the Virginia line experiment

The adaptive selective sweeps in the F_15_ generation of the AIL explained from 42 % to 51 % of the total additive genetic variance in this intercross, and we estimate that they contributed from 50 % to 92 % of the total additive genetic variance for 56-day body weight in the base population. The loci that were fixed for alternative alleles in the divergent lines after 40 generations of selection are thus likely have contributed a significant fraction of the selectable genetic variance present in the base population. This supports the theoretical expectation that standing variation will be an important contributor to the initial selection response for a population subjected to a novel selection pressure [26]. However, because standing variation was not exhausted even after an intense artificial long-term, single-trait selection, this suggests an adaptive value for standing variation over longer periods of time. This may be especially relevant in natural populations where selection is not as intense.

Although we have shown that standing genetic variation across 16 loci makes a major contribution to the additive genetic variance in the Virginia lines, other still undetected loci are also likely to have made an important contribution to the response up until generation 40 and thereafter. These will include standing genetic variants in the base population that were either present at lower allele frequencies, or have smaller genetic effects, and that were therefore not found in this study. Our results from evaluating the association between sweep length and genetic effects, as well as the distribution of genetic effects in the association analyses, suggest that at least some of the loci fixed at generation 40 merely had too low effects to be detected in the sample size of our current F_15_ generation. Further, the continued response to selection (Fig. 1), and the accompanying increase in fixation between the lines [3, 4], further supports this. Other contributions are expected from new mutations [26] and epistasis [27]. Although we have not explicitly explored the contributions by these types of mechanisms here, previous reports involving the Virginia lines have provided insights on these. For example, a major contribution has been reported from a network of interacting loci that via a capacitating epistatic mechanism was likely to have induced considerable selectable additive variation in response to selection [23, 24, 28, 29]. Moreover, a recent study of the within-line response to selection identified a novel allele that due to its rapid fixation within the high-weight selected line is likely to reflect selection on a newly arisen major adaptive allele [4]. Further work is, however, needed to quantify these sources of variation in relation to that of the standing variation.

## Conclusions

Here, we empirically confirm that the gradual response to long-term, bi-directional, single-trait selection in the Virginia chicken lines was, to a great extent, due to standing genetic variation across a highly polygenic genetic architecture in the common base population. A large number of loci, each having small allele-substitution effects, were major contributors to the selectable additive variance during adaptation. The loci that were fixed in the divergent lines after 40 generations of selection contributed much of the variation in the base population, thus providing empirical support for earlier work that has suggested that initial selection response is likely to result from selection on standing genetic variation. The considerably larger total additive genetic variance present within and between the lines after 40 generations of selection suggests that important contributions have also been made by a steady release of selectable genetic variation from, for example, standing genetic variants that increase in frequency during selection, complemented by new mutations and epistatic release of selectable additive genetic variance as shown in earlier studies of this population. In summary, these results provide not only novel insights to the genetics contributing to the gradual, continued, long-term response to selection in the Virginia lines, but also to the fundamental genetic mechanisms contributing to selection and adaptation. In particular, our empirical finding that fixation of several standing genetic variants within a highly polygenic genetic architecture has been an important dynamic feature during adaptation has important implications for further work on the genetics underlying adaptation.

## Methods

### Ethics statement

All procedures involving animals used in this experiment were carried out in accordance with the Virginia Tech Animal Care and Use Committee protocols.

### Animals and phenotyping

The animals used in this study were from an AIL bred from generation S40 parents from two lines of chickens divergently selected for juvenile body weight: the Virginia high (HWS) and low (LWS) lines. The HWS and LWS lines were founded in 1957 from a base population obtained by crossing seven partially inbred lines of White Plymouth Rock chickens. Since then, they have been subjected to bi-directional selection for a single trait, high or low 56-day body weight (BW56), respectively, and currently the lines have reached generation 58 (Fig. 1). More detailed information on the selected populations is available [10–12].

A complete description of the development of the Advanced Intercross population can be found in the publications describing the analysis of data from the F_2_-F_8_ generations of the AIL [29, 30]. The AIL (Fig. 1) was founded by F_0_ parents from generation S40 of the HWS and LWS lines, whose sex-average BW56 at that generation were 1,412 g (SE: ± 36 g) and 170 g (SE: ± 5 g), respectively. In total, 907 individuals were hatched in generation F_15_. Out of these, 852 survived until 56 days of age, when their body weight was measured. More details on the phenotypes of the founder lines and the AIL are provided in Table 3.

### DNA extraction

Blood samples were collected using a sterile needle and syringe, and then transferred to a tube containing disodium EDTA. DNA was extracted from whole blood samples using a Qiagen DNeasy kit.

### Marker selection and genotyping

In an earlier study, Johansson *et al.* [3] identified a large number of SNPs that were fixed for alternative alleles across the genome of the Virginia lines based on 60 K SNP chip genotypes obtained for individuals from generations 40 and 50 of the HWS and LWS lines. These SNPs were clustered into selective-sweep regions: 116 clusters containing 998 SNPs in generation 40 and 163 clusters with 1746 SNPs in generation 50. We selected 316 SNP markers to cover the 134 autosomal selective-sweep regions. As some markers were selected for fixation in generation 50, all were not fixed in the founders for the intercross obtained from generation 40. They were, however, all highly informative with an allele frequency difference ≥0.9 between LWS and HWS. Samples from all F_15_ birds with recorded 56-day weights (n = 852) were sent for genotyping at the 316 selected SNPs using the GoldenGate assay (Illumina, CA, USA) at the SNP technology platform in Uppsala (Sweden).

In total, 27 individuals and 64 SNPs failed to fulfill one or more of the following quality control criteria: call rate of individuals >0.9, call frequency of SNPs >0.9, and minor allele frequency >0.05. They were removed from the subsequent analysis, resulting in a final dataset consisting of genotypes for 252 SNPs in 825 birds. In total, the failed genotyping resulted in a loss of 26 initially targeted regions, resulting in a final coverage for 106 regions [3], 72 of which were present in both generation 40 and 50, and 34 emerged at generation 50.

### Re-clustering and summaries of targeted selective-sweep regions

After ordering the genotyped markers based on their physical locations on the galGal4 assembly, we redefined the clusters based on their genetic linkage in the F_15_ generation. This was to define clusters that segregated independently in the F_15_ generation in order to identify the number of independent, targeted selective sweeps that contribute to 56-day body weight in the lines. The criteria used for clustering markers were to assign adjacent markers that were no more than 50 cM apart into the same cluster and limit the range of each cluster to cover no more than 100 cM. According to these criteria, 99 independent regions were defined for use in the association analyses. The genetic distance between the markers in cM (Haldane mapping function) was estimated using the function est.map from the R/qtl package in R [31] where the function for an inbred intercross population could be used as the markers in this study are fixed, or nearly fixed, in the analysed population. Although a small number of the genotyped markers were not fixed for alternative alleles in the F_0_ founders, we decided that the obtained estimates of the genetic distances were sufficiently precise for re-clustering of the markers.

In total, we then screened for associations between 252 markers that passed quality control and 56-day body weight in 825 chickens from the F_15_ generation of the AIL. To estimate how many of the independently segregating regions contributed to 56-day body weight in the AIL F_15_ generation, we initially selected one representative marker from each of the 99 independently segregating clusters of markers, as defined above, in the F_15_ generation (Fig. 2; Additional file 1: Table S1) via a within-region, backward-elimination analysis. As mentioned above, these markers were located in 106 of the selective-sweep regions detected in the genome in two previous studies [3, 4].

### Heritability estimation

To estimate the heritability in the narrow sense for 56-day body weight, we used a linear mixed model:

$$ \mathrm{y}={\upbeta}_0+{\mathrm{w}\upbeta}_1+\mathrm{Zg}+\upvarepsilon $$

where y is the phenotype of 56-day body weight, *β*_*0*_ is an intercept term, *β*_*1*_ is the sex effect and *w* the associated indicator vector. Furthermore, the random polygenic effects *g* are normally distributed with correlation matrix given by the relationship matrix *A*, that is, a ~ N(0, *A*σ_A_^2^), *Z* is the associated incidence matrix, and *ε* is a normally distributed residual error ε ~ N(0, *I*σ_E_^2^). The relationship matrix *A* was constructed using the R/pedigree package [32]. The linear mixed model was fitted using the R/hglm package [33] and the heritability estimated as h^2^ = σ^2^_A_/(σ^2^_A_ + σ^2^_E_).

### Association analyses

#### Defining line origin for the genotyped marker alleles

The GoldenGate genotyping assay report F_15_ genotypes in an ‘ATCG’ basis without information about the line origin of the respective alleles. Therefore, we first transformed the genotypes into a ‘–1 0 1’ basis by comparing the genotypes in the F_15_ to that of the HWS/LWS F_0_ founders; ‘–1’ was used to represent the case when both alleles were of LWS line origin, ‘0’ that the individual was heterozygous, and ‘1’ that both alleles were of HWS line origin. In this way, positive estimates of the additive effects in the association analysis indicate that the HWS-derived allele increased weight, and negative additive effects that the LWS-derived allele increased weight (that is, that it is transgressive).

### Selection of markers to represent the independently segregating selective-sweep regions

The dataset was not sufficiently large to separate the effects of the linked markers within the 99 selective-sweep regions. Therefore, the first step in the association analysis was to simplify the subsequent analyses by selecting one marker in each of these regions to represent their joint effects in the subsequent multi-locus analysis. To select these markers, we used a within-locus backward-elimination approach in a linear model framework. The additive genetic effects of all markers in the selective-sweep region to be evaluated were then included together with the fixed effects of the sex for the bird. The analysis was thus based on the following model:

$$ \mathrm{y}={\upbeta}_0+{\mathrm{w}\upbeta}_1+{\mathrm{X}\upbeta}_2+\upvarepsilon $$

where *y* is the phenotype, *β*_*0*_ is an intercept term, *β*_*1*_ is the sex effect and *w* the associated indicator vector, *β*_*2*_ is the set of additive sweep effects modelled as fixed effects and *X* is the associated design matrix coded as −1, 0, 1 for the line origin of the marker genotypes, and *ε* is a normally distributed residual error. The number of markers within each region was limited and no problem of confounding between the fixed and random effects was detected.

Using backward elimination from the full model, we then identified the individual marker within each region that had the most significant effect, without requiring a particular significance for it at this stage of the analysis. This analysis was performed using custom written scripts in R.

### A multi-locus, backward-elimination analysis to identify adaptive selective-sweep regions

The objective of the confirmation study was to identify the set of selective-sweep regions that jointly contribute to 56-day body weight in the F_15_ generation of the AIL. The statistical analysis was chosen with the background knowledge that the genetic architecture of body weight in this population was highly polygenic [3, 4, 25, 30] and that potentially as many as half of the genotyped selective-sweep regions contributed to weight [3]. As all individuals in the AIL were progeny of dams of the same age, hatched on the same date, and reared separate from their parents, environmental contributions to between-family means in the F_15_ population may be considered minimal. Thus, a large portion of the difference in family means should be due to the joint effects of the many selective-sweep regions studied. When a large multi-locus mixed model was fitted to the data and fixed effects of markers across multiple selective-sweep regions were included together with a polygenic random effect in the model to account for family effects, there was a strong confounding between the fixed and random effects. This confounding may be explained by an assumption of linear mixed models being violated. A linear mixed model y = Xβ + Zu + e assumes that there is no correlation between a column in X, or a linear combination of several columns, with the true random effect Zu. This is a direct consequence of the basic assumption that the covariate and residuals in an ordinary linear model are independent. This might occur where the number of columns in *X* is large, and thus we deemed this analysis as unsuitable for use in the multi-locus analysis. However, as population structure might be of concern [34, 35], we chose to validate our results using a bootstrap-based approach developed for the same purpose for general deep-intercross populations, including Advanced Intercross Lines, by Valdar *et al.* [15]. These bootstrap analyses were implemented in custom scripts in the statistical software R [36].

The bootstrap based approach was implemented in a backward-elimination model-selection framework across the genotyped selective sweeps. Before doing the multi-locus analysis, we evaluated whether it was statistically appropriate to perform this analysis across all the 99 regions that segregated independently in the linkage analysis. For this, a standard measure to identify potential high-order collinearity, the ‘variance inflation factor’ or VIF, was used. Consistent with the linkage analysis, there was no large pair-wise correlation between the 99 markers. However, some marker genotypes could (almost) be written as a linear combination of the genotypes at all the other markers which would lead to collinearity problems in a multi-locus statistical analysis. Therefore the markers for the affected selective sweeps (21, 22, 80, 89, 90) were removed from the subsequent analyses.

The true size of the model (that is, the number of contributing regions) is unknown in advance and could range substantially from nearly half of the tested regions to a few individual associations. To compare models with such a wide range of variables to include, we opted to use an adaptive model selection criterion controlling the FDR [13, 14] developed for this purpose. The multi-locus models used during backward elimination were implemented in a standard linear model framework, starting with a full model including the fixed effects of sex and the additive effects of the 99 selected markers from the independently segregating regions. Convergence was based on two alternative FDR levels: 5 % and 20 %. The analysis was performed both in the original data, and using bootstrapping with 1,000 resamples. For the bootstrap analyses, the RMIP (Resample Model Inclusion Probability) was calculated for all regions in backward-elimination analyses with 5 % and 20 % FDR among the selected loci as termination criteria. A final model was decided for each FDR level by selecting the regions with RMIP >0.46 as suggested for an AIL generation F_18_ [15]. The additive genetic effects for each locus was estimated using the multi-locus genetic model described below including regions selected at 5 % and 20 % FDR.

To produce Fig. 3, the allele substitution effects were estimated for all markers selected at 20 % FDR. This was done in a multi-locus linear-model framework, where the genotypes of the markers in other significant regions selected in the same analysis were included as co-factors. The residual variances explained by the selected regions at 5 % and 20 % FDR were computed as the proportion of the residual variance of the null model including only the sex of the individual.

### Predicting the fraction of the HWS40-LWS40 line difference explained by the associated selective-sweep regions

Two alternative approaches were used to predict how much of the population difference of 1,242 g between the parental HWS and LWS from generation 40 (Table 2) could be explained by the selected regions at 5 % or 20 % FDR. This was done by regressing the individual’s phenotype to the total number of alleles of HWS origin carried across the evaluated regions. Using this approach, the standard error of the estimate for the average allele-substitution effect was lower than for the individual estimates of the loci. This analysis was performed across the regions selected at 5 % and 20 % FDR. The obtained estimates were then multiplied by the total number of allele substitutions between the HWS and LWS lines for this set of loci to obtain an estimate of the contribution to the line difference.

### Estimating the proportion of additive genetic variance in the base population explained by the significant selective sweeps

To estimate the contribution of the 16 significantly associated sweeps in the AIL F_15_ generation to the observed additive genetic variance in the base population for a particular set (θ) of allele frequencies at these loci, we calculated the total genetic variance explained for them as:

$$ {\upsigma_{\mathrm{A}}}^2\left(\uptheta \right)={\displaystyle \sum }2{\mathrm{p}}_{\mathrm{i}}{\mathrm{q}}_{\mathrm{i}}{\upalpha_{\mathrm{i}}}^2 $$

where σ_A_^2^(θ) is the total additive genetic variance across the 16 loci at the assumed allele frequencies, θ. ∑ is the sum over the 16 loci, and p_i_, q_i_, and α_i_ are the HWS and LWS allele frequencies and allele-substitution effects for each of the loci.

We evaluated two theoretical allele-frequency distributions across the 16 loci. First, when the adaptive, minor allele at each of the 16 sweeps was fixed in one of the partially inbred founder lines. Here, all the 16 individual allele-frequencies (p_i_) in θ were equal to 1/7. Second, when the adaptive alleles were randomly fixed across the seven founder lines. Here, the minor allele frequencies (p_i_) across the 16 loci were drawn randomly 10,000 times from a uniform distribution in the range of 1/7 to 6/7 to generate a set of θ from which average estimates of the additive genetic variance was calculated.

The proportions of the additive genetic variance in the base population contributed by the sweeps were then obtained by dividing the estimates obtained via the procedure described above with the product of the realised heritability and the total phenotypic variance observed in the fourth generation of the selection lines [37].

## Additional file

Additional file 1: Table S1. Table with additional information on the 252 genotyped markers and their clustering into independently segregating regions in the F_15_. (XLSX 68 kb)

## Abbreviations

AIL: Advanced Intercross Line
BW56: Chicken Body Weight at 56 days of age
GGA: Gallus Gallus Autosome
HWS: Virginia High Body-Weight Selected line
LWS: Virginia Low Body-Weight Selected line
